# Early In Vivo Osteogenic and Inflammatory Response of 3D Printed Polycaprolactone/Carbon Nanotube/Hydroxyapatite/Tricalcium Phosphate Composite Scaffolds

**DOI:** 10.3390/polym15132952

**Published:** 2023-07-05

**Authors:** Paulo Roberto Lopes Nalesso, Matheus Vedovatto, Julia Eduarda Schneider Gregório, Boyang Huang, Cian Vyas, Milton Santamaria-Jr, Paulo Bártolo, Guilherme Ferreira Caetano

**Affiliations:** 1 Graduate Program in Biomedical Sciences, University Centre of Hermínio Ometto Foundation, Araras 13607-339, SP, Brazil; paulorobertonalesso40@gmail.com (P.R.L.N.); matheusboiago@alunos.fho.edu.br (M.V.); julia.schneider@usp.br (J.E.S.G.); 2 Singapore Centre for 3D Printing, School of Mechanical and Aerospace Engineering, Nanyang Technological University, Jurong West, Singapore 639798, Singapore; boyang.huang@ntu.edu.sg (B.H.); cian.vyas@ntu.edu.sg (C.V.); pbartolo@ntu.edu.sg (P.B.); 3 School of Mechanical, Aerospace and Civil Engineering, The University of Manchester, Manchester M13 9PL, UK; 4 Graduate Program of Orthodontics, University Centre of Hermínio Ometto Foundation, Araras 13607-339, SP, Brazil; santamariajr@fho.edu.br; 5 Department of Social and Pediatric Dentistry, UNESP - São Paulo State University, Institute of Science and Technology - College of Dentistry, São José dos Campos 12245-000, SP, Brazil; 6 Division of Dermatology, Department of Internal Medicine, Ribeirão Preto Medical School, São Paulo University (USP), Ribeirão Preto 14049-900, SP, Brazil

**Keywords:** carbon nanotubes, ceramics, composites, inflammatory process, osteogenesis, tissue engineering, 3D printing

## Abstract

The development of advanced biomaterials and manufacturing processes to fabricate biologically and mechanically appropriate scaffolds for bone tissue is a significant challenge. Polycaprolactone (PCL) is a biocompatible and degradable polymer used in bone tissue engineering, but it lacks biofunctionalization. Bioceramics, such as hydroxyapatite (HA) and β tricalcium phosphate (β-TCP), which are similar chemically to native bone, can facilitate both osteointegration and osteoinduction whilst improving the biomechanics of a scaffold. Carbon nanotubes (CNTs) display exceptional electrical conductivity and mechanical properties. A major limitation is the understanding of how PCL-based scaffolds containing HA, TCP, and CNTs behave in vivo in a bone regeneration model. The objective of this study was to evaluate the use of three-dimensional (3D) printed PCL-based composite scaffolds containing CNTs, HA, and β-TCP during the initial osteogenic and inflammatory response phase in a critical bone defect rat model. Gene expression related to early osteogenesis, the inflammatory phase, and tissue formation was evaluated using quantitative real-time PCR (RT-qPCR). Tissue formation and mineralization were assessed by histomorphometry. The CNT+HA/TCP group presented higher expression of osteogenic genes after seven days. The CNT+HA and CNT+TCP groups stimulated higher gene expression for tissue formation and mineralization, and pro- and anti-inflammatory genes after 14 and 30 days. Moreover, the CNT+TCP and CNT+HA/TCP groups showed higher gene expressions related to M1 macrophages. The association of CNTs with ceramics at 10wt% (CNT+HA/TCP) showed lower expressions of inflammatory genes and higher osteogenic, presenting a positive impact and balanced cell signaling for early bone formation. The association of CNTs with both ceramics promoted a minor inflammatory response and faster bone tissue formation.

## 1. Introduction

Bone is a specialized mineralized tissue, mainly formed by non-collagenic proteins, type 1 collagen fibres, calcium, and phosphate ions, and is capable of repairing itself without external interventions. The maintenance and homeostasis of bone occur by remodelling, which is the substitution of damaged and/or senescent tissue during the lifetime of an individual [1,2]. During the bone repair process, the physiological inflammatory response is activated, aiming to unbridle the damaged tissue and cell recruitment (monocytes and neutrophils). Macrophages are substantial after their arrival and differentiation, and they initially have a pro-inflammatory profile (M1) secreting IL-6, IL-1, and tumoral necrosis factor-alpha (TNF-α). In sequence, they modify their role to an anti-inflammatory profile (M2) and release molecules, such as tumoral growth factor-beta (TGF-β) and IL-10, that are essential for tissue repair [3,4,5].

However, whenever the repair process is impaired, as in congenital defects, traumas, and the excision of tumours, the organism loses its ability to recover itself. Furthermore, some defects may evolve into critical defects, making an external intervention necessary [6,7,8]. Bone tissue engineering (BTE) researches the development, production, and applicability of materials as plausible treatments and/or tissue substitutes in bone repair as three-dimensional (3D) structures (scaffolds) via different techniques and approaches [9]. Additive manufacturing or 3D printing is a technique that allows controlled production of scaffolds layer-by-layer with specific architectures [10].

Polycaprolactone (PCL) is one of the synthetic polymers that is most studied within BTE. It is a biocompatible and degradable polymer that can be incorporated into other materials, aiming for the enhancement of its mechanical properties for bone repair. The association of PCL with ceramics, such as β-tricalcium phosphate (β-TCP) and hydroxyapatite (HA), could improve cell adhesion, proliferation, and differentiation [11]. Due to its similarities to native bone apatite and chemical composition, HA is a suitable material for bone regeneration, and it also presents properties of osteoconduction, bioactivity, and biocompatibility [12]. Similarly, β-TCP is another widely investigated material with osteoconductive and osteoinductive properties, and it also has a faster degradation rate compared to HA [13,14].

Previous research [15] has shown that the use of HA and TCP PCL-based composite scaffolds, whether individually or combined, enhanced bone repair when combined with electrical stimulation therapy (ES). Mineralization was greater with bone defects grafted with HA and TCP scaffolds after 30 days and was further enhanced after 60 days when ES was associated to the TCP scaffolds, with higher expressions of both *Runx-2* and *Osterix*. Although showing improved bioactivity, ceramic scaffolds also present limited mechanical properties, such as the resorption rate of HA and brittleness. Carbon nanotubes (CNTs) have exceptional mechanical and electrical properties that are highly relevant for bone applications given that the inclusion of CNTs can improve scaffold mechanics, which are important for load-bearing applications, and can be exploited to modulate bone cellular response through electrical/piezoelectrical stimulation. CNTs can mimic the extracellular matrix (ECM), and they have been demonstrated to contribute to higher cell adhesion, protein adsorption, and morphology regulation and differentiation, especially on neurons and osteoblasts due to their affinity to the binding proteins of cells [16,17,18].

A significant concern, though, is the cytotoxicity potential of CNTs and their ability to trigger the inflammatory process, which is dependent on the production method, CNT concentration, and chemical composition [19,20]. Our previous work using 3D printed PCL-based scaffolds containing 0.75 wt% and 3 wt% multi-walled CNTs showed no cytotoxicity, and it also showed enhanced mechanical properties, osteogenic differentiation, and bone formation [21]. However, no ceramic composite was considered. In addition to osteogenesis and mineralization, CNTs have been suggested to modulate the inflammatory response and macrophage phenotype, which can influence the tissue formation phase [22]. Patel [23], meanwhile, incorporated the CNT nanofibers into PCL, and reported a down-regulation effect on pro-inflammatory cytokines and macrophage recruitment. Mahon [24] investigated the use of scaffolds with nano-HA particles, and observed increases in the differentiation of macrophages into an anti-inflammatory phenotype (M2) by raising the production of IL-10.

In our previous work [25,26], comprehensive characterizations were performed on PCL/CNTs and PCL/CNTs/HA composites in terms of printability, morphology, physio-chemical properties, and biological properties. Multiple-walled carbon nanotubes were used. Rheological results suggested that the concentration of CNTs in a PCL matrix should be below 3 wt%, thereby ensuring the loss modulus of composite prevailing over the storage modulus at printing temperature (90 °C) in order to achieve better flowability and printability. Considering the similar thermal properties and biological properties (cell viability and proliferation) and reducing the risk of CNTs aggregations, 0.75 wt% of CNT concentration was selected to produce composite scaffolds. Porous scaffolds containing HA (20 wt%) and CNTs (0.75 wt%) were successfully printed using extrusion-based additive manufacturing, which can highly mimic the hierarchical nanostructure of native bone tissue with enhanced mechanical properties, cell proliferation, and osteogenic differentiation.

Although our previous work demonstrated the great potential of developed composites in vitro for bone tissue engineering and non-toxicity of using low concentration CNTs, the in vivo performances are unknown, particularly their inflammatory response regarding the use of CNTs. The primary aim of this work was to investigate the use of porous composite scaffolds for bone regeneration in terms of osteogenesis and inflammatory response. The evaluation could provide some evidence during the early stages of bone repair in order to determine the safety of low concentrations of CNTs and bioceramics considering both quantitative real-time PCR and histology.

## 2. Materials and Methods

### 2.1. Scaffolds Production

Composite scaffolds were 3D printed, as reported [25]. Briefly, PCL pellets (Perstorp Caprolactones, Cheshire, UK) were heated up to 90 °C for 20 min followed by the addition of CNTs, and were mixed for more 30 min to ensure homogenous dispersion, before adding HA nanoparticles (Sigma-Aldrich, St. Louis, MO, USA) and/or β-TCP microparticles (Sigma-Aldrich, St. Louis, MO, USA) in order to produce homogeneous mixtures. The specific compositions are shown in Table 1. The composite scaffolds were fabricated using a screw-assisted extrusion-based 3D printer (3D Discovery, REGENHU, Villaz-Saint-Pierre, Switzerland), considering the protocol used before [25]. Scaffolds were designed with a 0/90° lay-down pattern, 330 μm filament diameter, and 350 μm pore size [25]. The dimensions of the final produced scaffolds were 30 mm × 30 mm × 2.5 mm, but to fit into the calvaria of the animals, these scaffolds were further cut within the dimensions of 5 mm × 5 mm × 2.5 mm. Finally, the scaffolds were sterilized in 70% ethanol for 4 h, and then dried overnight.

### 2.2. Animals and Experimental Protocol

The in vivo experimental approach was approved by the ethical principles in animal research adopted by Hermínio Ometto Foundation’s Ethics Committee on Animal Use (024/2020). A total of 120 male Wistar rats (350 g, aged 3 months) were obtained from the Animal Facility of the University Centre of Hermínio Ometto Foundation (Araras, Brazil). The animals were divided as the treatment (scaffold) received in the critical defect (Table 1).

All of the animals were anesthetized with ketamine hydrochloride (30 mg/kg) and xylazine hydrochloride (10 mg/kg). The critical defect was created in the calvary bone (right parietal bone) by using an osteo I tip (PiezoHelse, Helse Dental technology, Santa Rosa de Viterbo, SP, Brazil), coupled with a dental ultrasound handpiece (Olsen, The Piezo Light D5 LED, Palhoça, SC, Brazil). A square critical defect (25 mm^2^) was created under a constant saline irrigation. The scaffolds were fitted exactly to the defect, without any physical fixation. The animals were randomly divided into the four experimental groups (PCL, CNT+HA, CNT+TCP, and CNT+HA/TCP). After the scaffold implantation, wounds were sutured with nylon 5-0 sutures (Shalon Medical, Goiânia, Brazil), followed by intraperitoneal and oral analgesic treatments using tramadol hydrochloride (1 mg/kg) and dipyrone (50 mg/kg), respectively, for 3 days. After 7, 14, 30, and 60 days, the animals were euthanized by anaesthetic overdose and the bone/scaffold samples were collected.

### 2.3. Histomorphometry

Histological analysis of bone tissue formation considering the mineralization area in the scaffolds was performed only at days 30 and 60 (n = 4 rats/experimental group) after surgery. In experimental periods before 30 days, such as 7 and 14 days, there is no mineralization to be assessed. The samples were fixed in a fixative buffer (10% formaldehyde) for 48 h, washed in water, and decalcified with aqueous 25% *w*/*v* sodium citrate solution and formic acid (ratio 1:1) for 60 days, with the solution being changed every 2 days. The samples were washed and dehydrated with an alcohol and xylol protocol, which consisted of bathing the samples in an increasing concentration sequence of these solutions, starting with 70% until 100%, and were then through two xylol solutions for maximum clarification of the samples; 1 h for each solution) before being embedded in paraffin (Paraplast, Histosec^®^, Merck KGaA, Darmstadt, Germany).

The samples were sliced into 4.0 µm cross-sections with a microtome (LEICA RM2245, Leica Microsystems, Wetzlar, Germany) and stained with Masson’s Trichrome. The histological sections were analysed by light-field microscopy (LEICA DM 4000 B, Leica Microsystems, Germany), and the connective and mineralized tissues were measured delimitating the respective areas. Briefly, ImageJ software (U.S. National Institutes of Health, Bethesda, MD, USA) was applied to these measurements, where a standard measurement for all photos was established (in μm^2^).

### 2.4. Gene Expression of Osteogenic and Inflammatory Response

The osteogenic and inflammatory responses were assessed at days 7, 14, 30, and 60 (n = 6 rats/experimental group) after surgery using reverse transcription quantitative polymerase chain reaction (RT-qPCR) analysis. The total RNA was extracted using TRIzol reagent (Invitrogen, Walthan, MA, USA), following the manufacturer’s instructions. Briefly, the bone/scaffold samples were macerated in liquid nitrogen and 1 mL of TRIzol was added. Cell lysis was performed using a homogenizer (Polytron System PT 1200 E, Kinematica AG, Lucerne, Switzerland). The concentration of RNA and the quality of the samples was measured using a spectrophotometer (ratios A260/280 and A260/230). The cDNA was synthesized from 1.5 µg of total RNA with the High-Capacity cDNA Reverse Transcription kit (Thermo Fisher Scientific, Waltham, MA, USA) according to the manufacturer’s instructions. The probes used for RT-qPCR are presented in Table S1 (Supplementary Information). The entire procedure was performed using the QuantStudio 3 Real-Time PCR Systems instrumentation platform (Thermo Fisher). The results were calculated using the 2^−ΔΔCt^ method.

### 2.5. Statistical Analysis

Experimental data are represented as mean ± standard error of the mean. Data were analysed using GraphPad Prism 8.0.2 software (GraphPad Software, San Diego, CA, USA). A Shapiro–Wilk normality test was conducted. One-way ANOVA with Tukey post hoc or Kruskal–Wallis with Dunn post hoc were applied. Significance levels were set at * *p* < 0.05; ** *p* < 0.01; *** *p* < 0.005; **** *p* < 0.0001.

## 3. Results

### 3.1. Osteogenic Gene Expression

**Figure 1:**
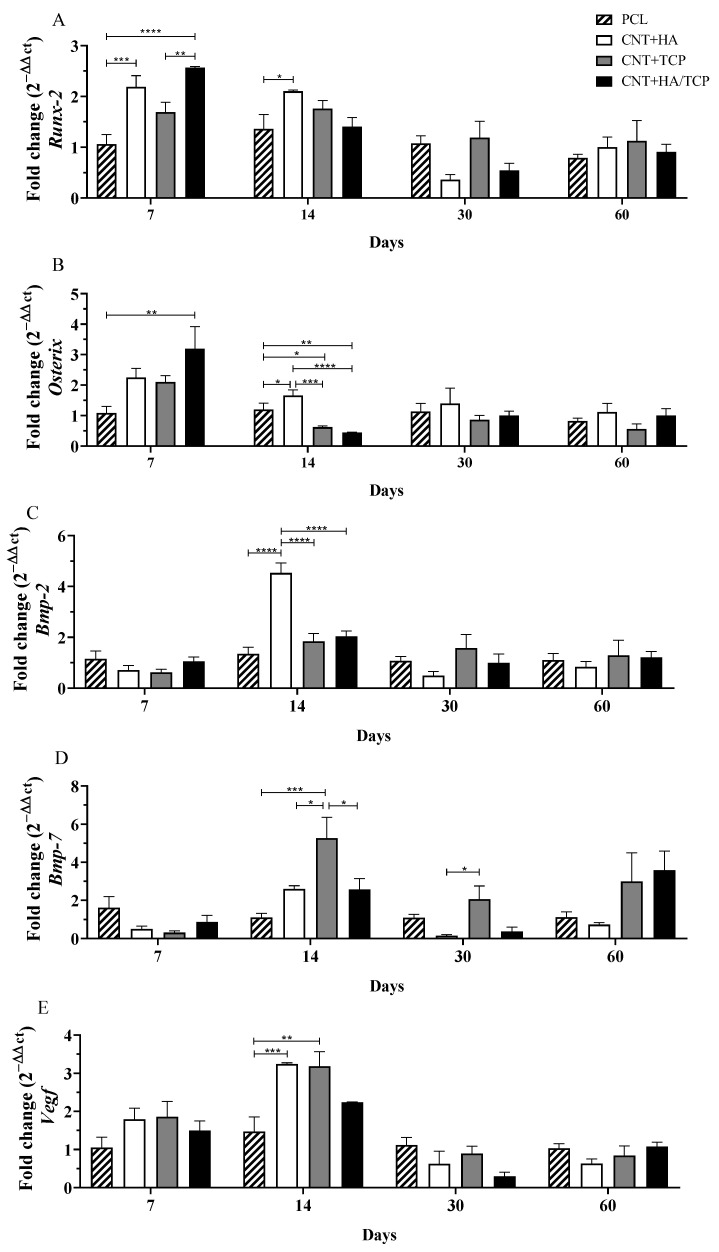
Osteogenic gene expression (qPCR) showing the fold change for (**A**) *Runx-2*, (**B**) *Osterix*, (**C**) *Bmp-2*, (**D**) *Bmp-7*, and (**E**) *Vegf* at days 7, 14, 30 and 60 in PCL, CNT+HA, CNT+TCP, and CNT +HA/TCP. * *p* < 0.05, ** *p* < 0.01, *** *p* < 0.001, **** *p* < 0.005.

*Runx-2* has been demonstrated to promote mesenchymal stem cell (MSC) differentiation into immature osteoblasts, whilst *Osterix* is a marker for mature osteoblasts and promotes osteogenesis [27]. The CNT+HA/TCP group presented the highest *Runx-2* expression on the seventh day, which was significantly higher when compared to the PCL group (2-fold change) and the CNT+TCP group (1.5-fold change) (Figure 1A). Furthermore, the CNT+HA group presented higher expression compared to PCL (2-fold change) on the 7th and 14th days. A decreasing trend in *Runx-2* expression was observed in all groups from day 14––similar gene expression with no important difference among them.

The CNT+HA/TCP group presented significantly higher *Osterix* expressions compared to PCL (3-fold change) at day 7 (Figure 1B). The CNT+HA and CNT+TCP groups presented a 2-fold higher expression than PCL. On the 14th day, the CNT+HA group presented significantly higher expression compared to all groups. The CNT+TCP and CNT+HA/TCP groups showed reduced expression compared to the PCL group. After day 30 and 60, no significant difference was observed among the groups, with overall reduced expression.

No significant difference was observed in *Bmp-2* expression among all of the groups after 7 days (Figure 1C). However, on the 14th day, the CNT+HA group expressed significantly higher expression than the other groups (4-fold change)**.** After day 30 and 60, no significant difference was observed among the groups, with overall reduced expression. Similar to *Bmp-2*, the expression of *Bmp-7* also showed no increase at day 7 (Figure 1D). However, on the 14th day, the CNT+TCP group presented significantly higher expression than the PCL (5-fold change), CNT+HA (2-fold change), and CNT+HA/TCP (2-fold change) groups. After 30 days, the CNT+TCP group (2-fold change) still presented high expression, which was different to the CNT+HA group (reduced expression). At day 60, no significant difference was observed, despite the CNT+TCP and the CNT+HA/TCP groups both presenting higher expression (3-fold change).

Regarding to *Vegf* gene expression (Figure 1E), at day 7, all groups had similar expression (1.5-fold change) compared to PCL. At day 14, the CNT+HA and CNT+TCP groups presented similar results, both higher than the PCL group (3-fold change), followed by the CNT+HA/TCP one (2-fold change). Reduced *Vegf* expression was observed in all groups on the 30th and 60th days––similar gene expression with no important difference among them.

Considering *Runx-2* and *Osterix* (osteogenic genes), the CNT+HA, CNT+TCP, and CNT+HA/TCP groups all demonstrated higher expression compared to the PCL group on the 7th day, with evidence to CNT+HA/TCP. On the 14th day, the CNT+HA group showed important *Bmp-2* stimulation, while the CNT+TCP group showed *Bmp-7* on the 14th and 30th days.

### 3.2. Inflammatory and Macrophage Response

**Figure 2:**
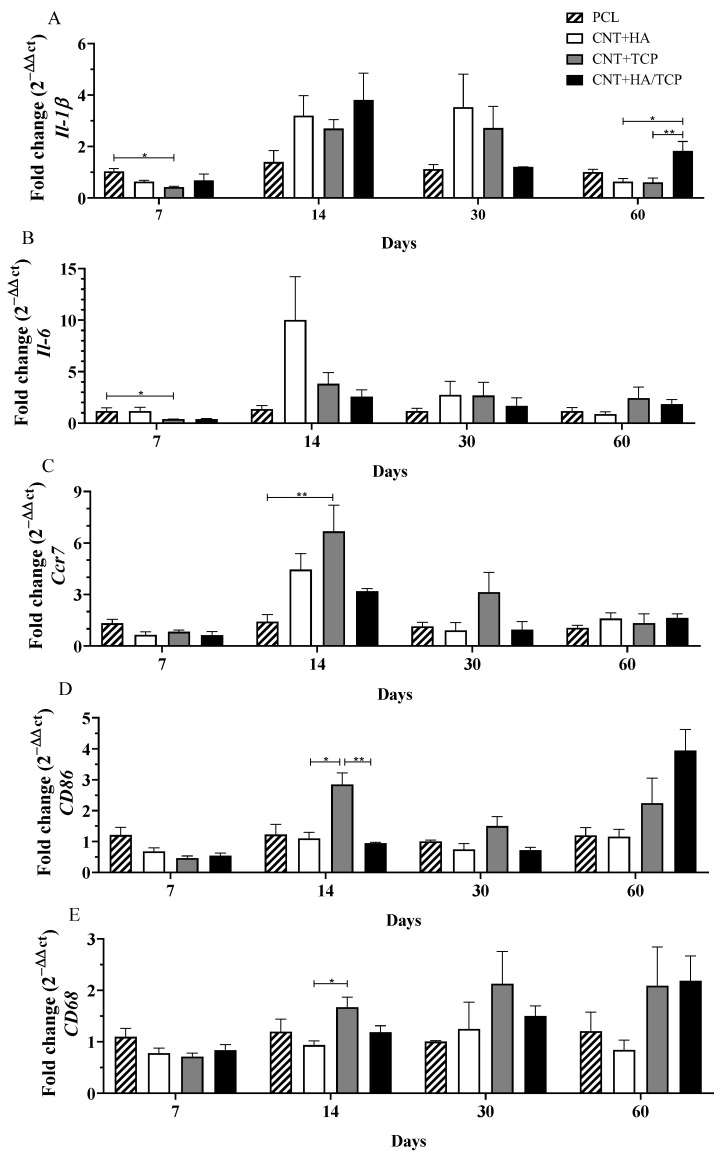
Inflammatory gene expression (qPCR) showing the fold change for (**A**) *Il-1β*, (**B**) *Il-6*, (**C**) *Ccr7*, (**D**) *CD86*, and (**E**) *CD68* at days 7, 14, 30, and 60 in PCL, CNT+HA, CNT+TCP, and CNT+HA/TCP. * *p* < 0.05, ** *p* < 0.01.

**Figure 3:**
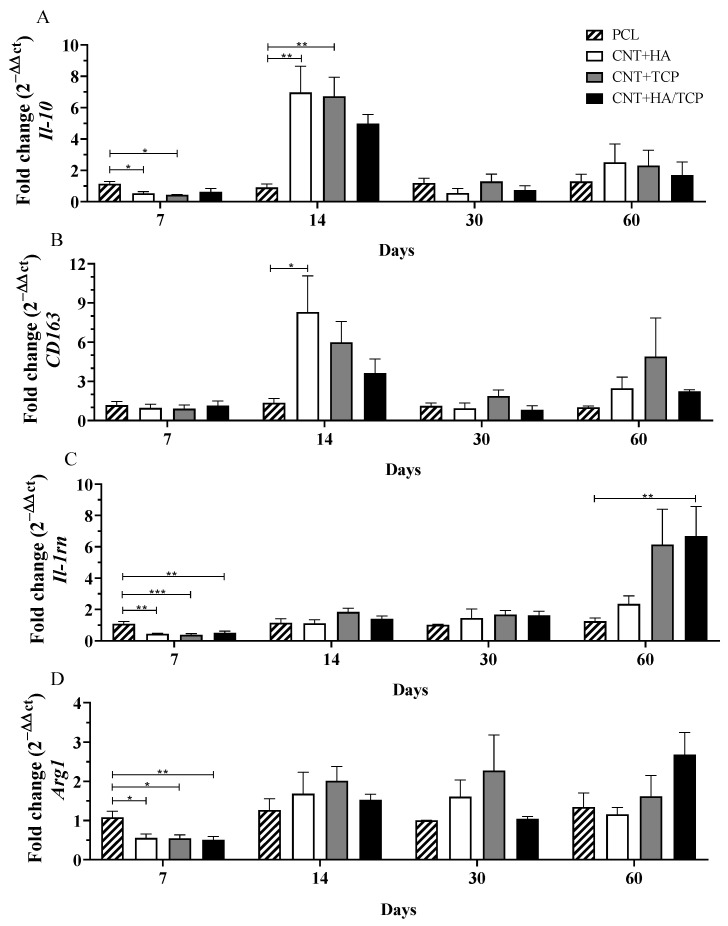
Anti-inflammatory gene expression (qPCR) showing the fold change for (**A**) *Il-10*, (**B**) *CD163*, (**C**) *Il-1rn*, and (**D**) *Arg1* at days 7, 14, 30, and 60 in PCL, CNT+HA, CNT+TCP, and CNT+HA/TCP. * *p* < 0.05, ** *p* < 0.01, *** *p* < 0.001.

The inflammatory response and changes in macrophage phenotype are important factors in the biocompatibility and long-term success of an implant. The results are presented as pro-inflammatory (Figure 2) and anti-inflammatory factors (Figure 3).

#### 3.2.1. Pro-Inflammatory Phase

The gene expression of the pro-inflammatory interleukins, *Il-1β* and *Il-6*, and M1-phenotye macrophages surface markers *Ccr7*, *CD86*, and *CD68* gene expression over the experimental period are presented in Figure 2. At day 7, the CNT+TCP group presented significantly lower *Il-1β* expression (0.5-fold change) compared to the PCL group (Figure 2A). At day 14, even though there was no evidence of difference, all three of the CNT-composite groups presented higher expressions than the PCL one, ranging from a 2.5-fold change (CNT+TCP) to a 3.5-fold change (CNT+HA/TCP). A similar expression to day 14 was observed on day 30, but with reduced expression in the CNT+HA/TCP group. By day 60, the CNT+HA/TCP group showed a 2-fold change compared to PCL and a significantly higher expression than the CNT+HA and CNT+TCP groups. At day 7, similar to *Il-1β* gene expression, the CNT+TCP group presented significantly lower *Il-6* expression (0.5-fold change) compared to the PCL group (Figure 2B). By day 14, CNT+HA presented ~10-fold higher expression than PCL but no significant difference. At day 30 and day 60, all groups presented similar expression.

*Ccr7* and *CD86* are M1-phenotype macrophages’ surface markers, while *CD68* acts as a surface marker for both M1 and M2-phenotype macrophages. On the 7th day, all groups presented similar expression of those genes. However, after 14 days (Figure 2C), the CNT+TCP group presented the highest (~6.5-fold change) and was statistically different from the PCL group, followed by the CNT+HA and CNT+HA/TCP groups (4-fold and 3-fold change, respectively). At the day 30, despite the absence of statistically significant difference, the CNT+TCP group presented a 3-fold change expression compared to the PCL group. On the 14th day, (Figure 2D) the CNT+TCP displayed higher *CD86* expression (almost 3-fold change), which was statistically different to the CNT+HA and CNT+HA/TCP groups (both 1-fold change). After day 30, no important difference was observed, while after 60 days, despite no evidence of difference, the CNT+TCP and CNT+HA/TCP groups presented higher *CD86* expression (2 and 4-fold change, respectively) compared to both the CNT+HA and the PCL groups. After 14 days (Figure 2E), the CNT+TCP group presented higher *CD68* expression, which was statistically different to CNT+HA. Similar to *CD86*, after 60 days, and despite no evidence of difference, the CNT+TCP and CNT+HA/TCP groups presented higher expression (2-fold change) compared to both the CNT+HA and the PCL groups.

#### 3.2.2. Anti-Inflammatory Phase

The gene expression of the anti-inflammatory interleukin *Il-10* and the M2-phenotye macrophages surface markers *CD163*, *Il-1rn*, and *Arg1* are presented in Figure 3. Figure 3A shows that all three composite groups presented reduced *Il-10* compared to the PCL group. On the other hand, on the 14th day, all three composite groups presented increased *Il-10* compared to the PCL one, with evidence of difference to the CNT+HA and CNT+TCP groups (both 6-fold change). The CNT+HA/TCP group presented a 5-fold change, but no significance was found. After 30 and 60 days, no important difference was observed among the groups.

The CNT+HA group presented *CD163* gene expression 8-fold change higher than the PCL on the 14th day (Figure 3B), followed by CNT+TCP (6-fold change) and CNT+HA/TCP (4-fold change). After 30 and 60 days, no important difference was observed among the groups. Regarding *Il-1rn* (Figure 3C), it is possible to notice that all three of the composite groups presented significantly reduced gene expression compared to the PCL one. On the 14th and 30th days, all four groups presented reduced and similar expression, but on the 60th, the CNT+HA/TCP group present statistical difference to the PCL one (7-fold change), followed by the CNT+TCP (6-fold change) and the CNT+HA groups (2-fold change), respectively. Arginase 1 (*Arg1*) is a M2a surface marker and its results revealed that after 7 days (Figure 3D), similar to *Il-1rn*, all three composite groups presented significantly reduced gene expression compared to PCL. On the 14th and 30th days, all four groups presented similar expression. On the 60th day, only the CNT+HA/TCP showed higher gene expression when compared to the PCL group, with, approximately, a 2.5-fold change, but with no evidence of difference.

### 3.3. Tissue Formation

**Figure 4:**
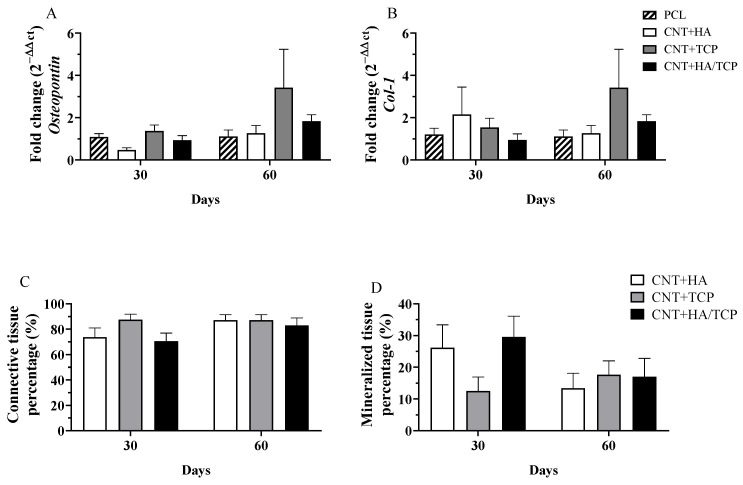
Gene expression (qPCR) showing the fold change for (**A**) *Osteopontin*, (**B**) *Collagen I*; and histomorphometry for (**C**) connective tissue percentage, and (**D**) mineralized tissue percentage at days 30 and 60 in PCL, CNT+HA, CNT+TCP, and CNT+HA/TCP.

Tissue maturation and mineralization is an essential physiological process to regenerate bone tissue, and its absence could result in impaired tissue recovery. Figure 4A,B show the results for *osteopontin* (*OPN*) and *collagen I* gene expression, respectively, over the experimental period. No evidence of statistical difference was found. However, higher *OPN* and *collagen I* expression in the CNT+TCP (3-fold change) and CNT+HA/TCP (1.5-fold change) groups than the PCL one was observed after 60 days. The histological evaluation shows the percentage of connective and mineralized tissue observed in the groups (Figure 4C,D). There was no difference in connective tissue formation, since all groups had similar production after 30 and 60 days. However, on the 30th day, the CNT+HA and CNT+HA/TCP groups showed higher percentages of mineralized tissue compared to the CNT+TCP one. On the 60th day, a slightly difference was observed between the CNT+TCP and CNT+HA/TCP groups compared to the CNT+HA one.

**Figure 5:**
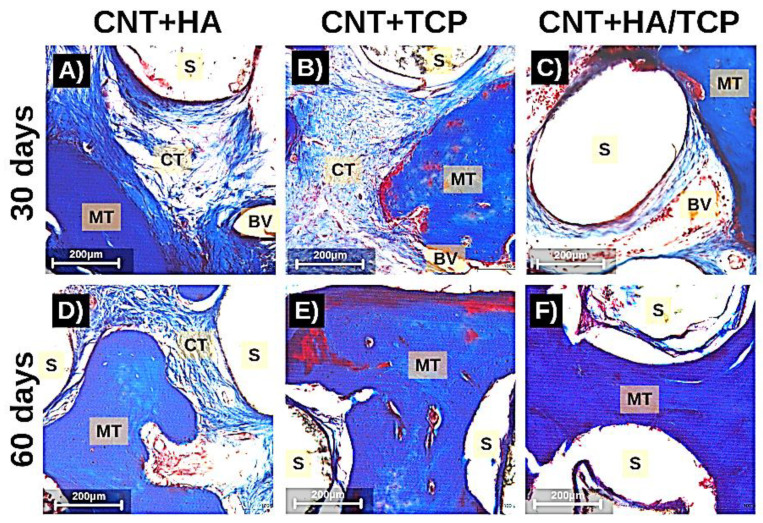
Photomicrography stained with Masson Trichrome (200× magnification) showing the tissue formation in the scaffolds after 30 days (**A**–**C**) and 60 days (**D**–**F**). MT: mineralized tissue; CT: connective tissue; BV: blood vessel; S: scaffolds fibres (scale = 200 µm).

Moreover, Figure 5 shows images of histological samples stained with Masson’s Trichrome and how tissue organized itself during the repair process (30 and 60 days). It can be observed that there were less connective tissue fibres on the images from the CNT+HA/TCP group after 30 days, whilst the mineralized tissue was present during both of the periods (30 and 60 days). The scaffold fibres (S) can be observed (white circles), and so can connective tissue (CT in light blue fibrous-like tissue), osteoid/mineralized tissue (MT in dark blue tissue), and blood vessels (BV).

## 4. Discussion

The combination of CNTs and bioceramics in the PCL-based scaffold is hypothesized to enhance the expression of genes related to mesenchymal stem cell differentiation towards osteoblast lineage and the maturation of osteoblasts. The *Runx-2* and *Osterix* gene expression demonstrated the expected effect, especially in the CNT+HA/TCP group, which could lead to faster and more efficient tissue formation, as can be seen in the literature [27,28,29,30].

Curiously, an increased *Bmp-2* expression in the CNT+HA group and an increased *Bmp-7* in the CNT/TCP group on the 14th day was observed. [15] reported similar data with the use of PCL scaffolds with HA 20 wt%. Even though the CNT+HA/TCP group was created with HA 10 wt% and TCP 10 wt%, the same effect was not observed for these two genes. It was also reported in vitro [31] that the association of CNTs to PCL+HA scaffolds promoted higher expressions of COL-1, while previous reports in in vivo studies found faster tissue growth and mineralization, and these also corroborate our data, most probably due to the osteoconduction property [29,30,31,32,33]. *Bmp-7* is also associated with matrix remodelling by osteoblasts. The higher expression by treatment with CNT+TCP and CNT+HA/TCP suggests that these groups could have achieved a faster remodelling phase for bone maturation.

Our data also suggests that higher concentrations of ceramics (20 wt%) enhanced the expression of *Vegf* in comparison to ceramics at lower concentrations (10 wt%), promoting a faster vascularization and cellular influx. As previously observed [21], higher vascularization was found treating bone defects with PCL/MWCNTs in comparison to those with no scaffolds, which suggests that the presence of the scaffold led to faster endothelial cell adhesion, and which also provided an anchorage point for these cells. As a result, for the implantation of the scaffolds, the cells from the immune system, especially those from the innate response (such as macrophages and neutrophils), arrive rapidly to the site of injury, releasing cytokines and chemical factors as endothelial cells as well, which, after the angiogenesis has started, will provide the site with new nutrients and new growth [34]. The CNT/HA and CNT/TCP groups presented higher gene expression for pro-inflammatory and anti-inflammatory markers, which could be influenced by faster vascularization. However, the CNT/HA+TCP group presented high expression for both, although they maintained a sort of balance between the two phases of inflammation. The pro-inflammatory and anti-inflammatory gene expression suggests that an essential inflammatory response was engaged, but that it was balanced. The use of 20 wt% of ceramics seemed to invoke such responses at early stages, as the CNT+HA/TCP group showing the opposite at late stages. Once more, no drawbacks by using PCL/MWCNTs in vivo at low concentrations [21]. Another study [35] has reported no signs of toxicological nor inflammatory responses whilst applying CNT+HA into the liver and kidney of rabbits, and this demonstrate that despite its effects on bone repair, TCP can also create an inflammatory response, albeit a controlled one. Accordingly, the two groups containing TCP on their composition presented an expressive expression of macrophage polarization markers for both the M1 phenotype and the M2 phenotype.

Prior to our investigation, [15] found greater tissue formation in PCL+HA scaffolds after 30 days, whilst PCL+TCP presented higher tissue formation after 60 days using scaffolds produced with 20 wt% of ceramics for each one. As demonstrated in our study, by incorporating both HA and TCP at 10 wt% in CNT and PCL scaffolds, a faster mineralization could be reached that also takes into account the higher osteogenic expressions in early stages of our study. Moreover, it is also possible suggest the lack of HA in the CNT+TCP group could be the cause of less mineralized tissue after 30 days when compared to other groups. Another study [36] synthesized a MWCNT-HA scaffold in order to coat a pure titanium surface, and observed great HA crystal formation on the surface with MWCNTs. This corroborates with our histological data regarding the greater percentage of new mineralized tissue in the groups with HA incorporated. However, since β-TCP is able to induce apatite formation, and, thus, elevated HA formation [37], our data suggested quite the opposite—namely, that it was the lack of HA as opposed to β-TCP that most affected the mineralized tissue formation.

## 5. Conclusions

This study investigated the early in vivo osteogenic and inflammatory response of 3D printed PCL-based composite scaffolds containing CNTs, HA, and TCP in order to evaluate both early osteogenesis and inflammatory responses in a bone critical defect rat model. Due to its higher stiffness, CNTs may have contributed to a higher endurance of the scaffolds, allowing the ceramic materials to be well absorbed. The CNT+HA and CNT+TCP groups presented higher expressions of genes related to osteogenesis (tissue formation and mineralization), and, in addition, both pro- and anti-inflammatory stimulus, thereby showing the benefits of higher concentrations of ceramics (20 wt%). However, the CNT+HA/TCP group expressed osteogenic genes early on the 7th day and less inflammatory markers throughout the whole experiment period, which demonstrates that even lower concentrations of ceramics (10 wt%) might have an influence on tissue formation and mineralization, and might also have an influence on the modulation of inflammatory events by CNTs. Further studies are required in order to gain a clearer view of what is truly happening concerning macrophage polarization and how the ceramics trigger an inflammatory response.

## Figures and Tables

**Table 1:** Composition of 3D printed scaffolds for in vivo evaluation of bone regeneration.

Scaffolds	Material Ratio in Percentage Weight (wt%)
**PCL**	PCL (100 wt%wt%)
**CNT + HA**	PCL (79.25 wt%); CNTs (0.75 wt%); HA (20 wt%)
**CNT + TCP**	PCL (79.25 wt%); CNTs (0.75 wt%); TCP (20 wt%)
**CNT + HA/TCP**	PCL (79.25 wt%); CNTs (0.75 wt%); TCP (10 wt%); HA (10 wt%)

## Data Availability

Data available on request from the corresponding author due to privacy and also ethical issues.

## Supplementary Materials

The following supporting information can be downloaded at: https://www.mdpi.com/article/10.3390/polym15132952/s1, Table S1. TaqMan assays.
