# Supplementary material for: Early In Vivo Osteogenic and Inflammatory Response of 3D Printed Polycaprolactone/Carbon Nanotube/Hydroxyapatite/Tricalcium Phosphate Composite Scaffolds
Source: Polymers (Basel). 2023 Jul 5;15(13):2952. doi: 10.3390/polym15132952 (PMC10346620; doi:10.3390/polym15132952)
Supplement: Supplementary file 1 [file polymers-15-02952-s001.zip › polymers-2475277-supplementary.pdf]

Supplementary material

Table S1. TaqMan assays.

Genes	Biological function	Codes
Gapdh	Glycolysis pathway's enzyme	Rn01775763_g1
Runx-2	MSC differentiation into immature osteoblasts	Rn01512298_m1
Osterix	Expressed by mature osteoblasts, promotes osteogenesis	Rn02769744_s1
Bmp-2	Bone formation	Rn00567818_m1
Bmp-7	Bone formation and angiogenesis	Rn01528889_m1
Vegf	Blood vessels formation stimulation	Rn01511602_m1
Il-6	Organism defence during the acute inflammation process and tissue lesion	Rn01410330_m1
Il-1β	Pro-inflammatory cytokine secreted by macrophages	Rn01460205_m1
Ccr7	Chemokine (C-C motif) receptor 7; M1-macrophage phenotype	Rn02758813_s1
CD86	M1-macrophage phenotype surface marker	Rn00571654_m1
CD68	Pan-macrophage phenotype surface marker	Rn01495634_g1
Il-10	Inflammation control and modulation of the adaptative immune response	Rn01483988_g1
Il-1rn	Interleukin 1 receptor antagonist; M2-macrophage phenotype	Rn02586400_m1
CD163	M2-macrophage phenotype surface marker	Rn01492519_m1
Arg1	Arginase 1; M2a-macrophage phenotype	Rn00691090_m1
